# Combining hot-compressed water and ball milling pretreatments to improve the efficiency of the enzymatic hydrolysis of eucalyptus

**DOI:** 10.1186/1754-6834-1-2

**Published:** 2008-04-15

**Authors:** Hiroyuki Inoue, Shinichi Yano, Takashi Endo, Tsuyoshi Sakaki, Shigeki Sawayama

**Affiliations:** 1 Biomass Technology Research Center, National Institute of Advanced Industrial Science and Technology (AIST), 2-2-2 Hiro-Suehiro, Kure, Hiroshima 737-0197, Japan

## Abstract

**Background:**

Lignocellulosic biomass such as wood is an attractive material for fuel ethanol production. Pretreatment technologies that increase the digestibility of cellulose and hemicellulose in the lignocellulosic biomass have a major influence on the cost of the subsequent enzymatic hydrolysis and ethanol fermentation processes. Pretreatments without chemicals such as acids, bases or organic solvents are less effective for an enzymatic hydrolysis process than those with chemicals, but they have a less negative effect on the environment.

**Results:**

The enzymatic digestibility of eucalyptus was examined following a combined pretreatment without chemicals comprising a ball milling (BM) and hot-compressed water (HCW) treatment. The BM treatment simultaneously improved the digestibility of both glucan and xylan, and was effective in lowering the enzyme loading compared with the HCW treatment. The combination of HCW and BM treatment reduced the BM time. The eucalyptus treated with HCW (160°C, 30 minutes) followed by BM (20 minutes) had an approximately 70% yield of total sugar with a cellulase loading of 4 FPU/g substrate. This yield was comparable to the yields from samples treated with HCW (200°C, 30 minutes) or BM (40 minutes) hydrolyzed with 40 FPU/g substrate.

**Conclusion:**

The HCW treatment is useful in improving the milling efficiency. The combined HCW-BM treatment can save energy and enzyme loading.

## Background

Lignocellulosic biomass, such as wood and agricultural residues, is an attractive material for fuel ethanol production because it contains large amounts of potentially fermentable sugars in the form of cellulose and hemicellulose. Recent studies have indicated that the conversion of lignocellulosic biomass to ethanol involves three processes: (1) a pretreatment process to increase the digestibility of cellulose and hemicellulose in the feedstock; (2) an enzymatic hydrolysis process to recover fermentable sugars from the pretreated material; and (3) a fermentation process to convert the obtained sugars into ethanol [1]. The cost of ethanol production from lignocellulosic biomass is too high based on current technologies and, therefore, considerable research effort has been devoted to improving the hydrolysis of the feedstock [2].

The choice of pretreatment for lignocellulosic biomass has a major influence on the cost of subsequent enzymatic hydrolysis [3-5]. The purpose of the pretreatment is to alter or remove the physical and chemical impediments that inhibit the accessibility of the enzyme to the substrates. The removal of hemicellulose and lignin from lignocellulosic biomass by adding inexpensive chemicals such as acids, bases or organic solvents is known to have a significant effect on enzymatic hydrolysis [2,5-7]. However, these chemicals may require corrosion-resistant reactors and must be neutralized or recycled to reduce their negative impacts on the environment as well as on subsequent processes (for example, fermentation).

Uncatalyzed hydrothermal hydrolysis and mechanical comminution of lignocellulosic biomass are regarded as environmentally benign processes because no chemicals are used. Hydrothermal treatment using steam or hot-compressed water (HCW) is an effective process for the enzymatic hydrolysis of hardwoods and agricultural residues, although it has a relatively small effect on softwoods [1,2,8,9]. Water itself and the acetyl groups in hemicellulose act as acids at around 200°C, thereby dissolving both hemicellulose and lignin from lignocellulosic biomass [10-15]. Mechanical comminution is sometimes required to make material easier to handle through subsequent processes [1,16]. Furthermore, reduction of the crystallinity of cellulose by ball milling (BM) or a compression milling treatment has been reported to increase the enzymatic digestibility of various cellulosic substrates including softwoods remarkably [17-21]. The efficiency of enzymatic hydrolysis is greatly affected by the degree of milling, which consumes excessive amounts of energy [20-22].

Here we report the effects of a combined HCW-BM treatment on the enzymatic hydrolysis of eucalyptus. This pretreatment technology was designed to shorten the milling time of BM treatment by using a HCW-treated residue. The optimized HCW-BM treatment has the possibility of reducing not only the energy requirement for each pretreatment but also the cellulase loading for enzymatic hydrolysis.

## Results and discussion

### Effect of BM treatment on the enzymatic digestibility of eucalyptus

In this study, sugar yields reported were calculated on the basis of the sugar contents in the dried eucalyptus: 40.0% glucan, 10.4% xylan and a total of 50.4% sugars. The enzymatic digestibilities of pretreated materials were evaluated using an enzyme cocktail containing Acremonium cellulase from *Acremonium cellulolyticus*, which is a well-known commercial cellulase that has higher β-glucosidase activity than that of conventional cellulase from *Trichoderma *species [23].

The glucan and xylan digestibility of BM-treated eucalyptus was evaluated for various milling times (10-120 minutes). The crystallinity index (CrI) of eucalyptus pretreated using a planetary BM decreased to 7.6% with 20 minutes of milling (Table 1), indicating that cellulose microfibrils in the wood cell wall were disrupted. However, the material milled for 20 minutes appears showed an insufficient improvement in the enzymatic digestibility, because only 46.8% glucan and 34.3% xylan were hydrolyzed with 40 FPU/g substrate. The enzymatic digestibilities of both glucan and xylan were significantly elevated by a prolonged milling time (120 minutes), although the CrI of this material was almost the same as that of the material milled for 20 minutes (Table 1). The mechanical energy generated by milling is known to cause some mechanochemical effects in solid-state materials [24]. Our results suggest that the effect of BM treatment on the enzymatic digestibility is closely related to the mechanochemical breakdown of an ultrastructure such as the cellulose-hemicellulose-lignin network that occurs following the disruption of cellulose microfibrils.

**Table 1:** Effect of milling time on enzymatic digestibility of eucalyptus in ball-milling treatment

Milling time (minutes)	Yields of hydrolyzed sugars		Total yield (%)	CrI^a ^(%)
			
	Glucose (%)	Xylose (%)		
0	-	-	-	59.7
10	16.0	20.5	16.9	25.9
20	46.8	34.3	44.2	7.6
40	70.4	49.2	66.0	3.0
120	89.7	72.5	86.2	6.4

The ball-milling (BM)-treated sample (1%) was hydrolyzed with an enzyme cocktail (40 FPU/g substrate) for 72 hours as described in materials and methods. BM treatment was performed using pulverisette 7. The hydrolysis yields of glucose and xylose are calculated based on the glucan and xylan contents, respectively, in the dry weight of untreated biomass. Total yield of hydrolyzed sugars is calculated from the combined contents of glucan and xylan in the dry weight of untreated biomass. ^a^Crystallinity index of the BM-treated sample.

**Figure 1:**
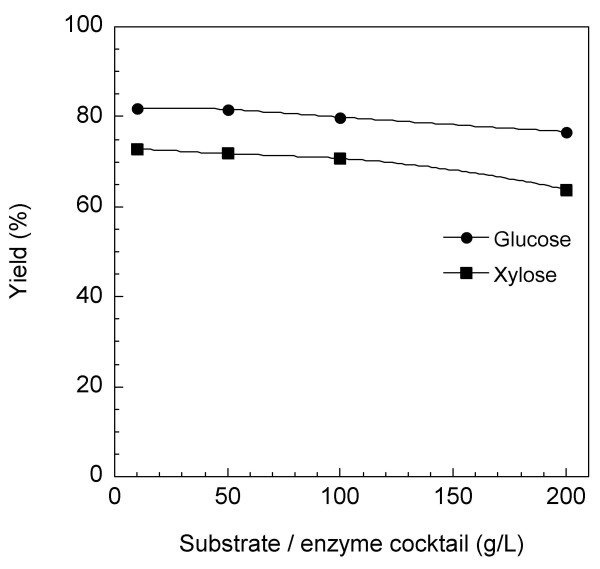
**Effect of substrate concentration on the enzymatic digestibility of eucalyptus treated with ball milling**. To prepare a large quantity of the sample, ball-milling (BM) treatment (240-minute milling) was performed using pulverisette 5 as described in materials and methods. Various amounts of substrates were mixed and hydrolyzed for 72 hours with the same volume of enzyme cocktail (4 FPU/g substrate). Yields of hydrolyzed glucose and xylose are expressed as a percentage of the theoretical amount based on the glucan and xylan contents, respectively, in the dry weight of untreated biomass.

The enzymatic digestibility of the BM-treated eucalyptus was hardly affected by the substrate concentration regardless of the presence of a high lignin content. The digestibility of glucan and xylan and their total yield were estimated to be 76.7%, 63.9% and 74%, respectively, even at a substrate concentration of 200 g/l of the enzyme cocktail containing 4 FPU/g substrate (Fig. 1). The concentrations of glucose and xylose produced in this hydrolysate were 68 g/l and 15 g/l, respectively. The advantages of using a high concentration of substrate are that: (1) the capital equipment cost for hydrolysis can be reduced; and (2) rich sugar syrups can be produced and consequently the costs of ethanol fermentation and distillation are reduced [20]. We furthermore confirmed that the glucose in the hydrolysate was converted to ethanol by baker's yeast and that the yield was above 90% of the theoretical within 24 hours (data not shown). This suggests that the BM-treated eucalyptus has no inhibitory effect on the enzymatic hydrolysis or the ethanol fermentation.

### Enzymatic digestibility of xylan in BM-treated eucalyptus

A supplemental hemicellulase, Optimash BG, had no effect on increasing glucan digestibility of 120-min milled eucalyptus in the presence of excess cellulase (40 FPU/g substrate), while it had great influence on increasing xylan digestibility. In the enzyme cocktail containing 40 FPU/g substrate of Acremonium cellulase, xylanase activity from Acremonium cellulase (1267 U/g substrate) was 25-fold higher than that from Optimash BG (51 U/g substrate). However, the supplementation of Optimash BG to the enzyme cocktail caused an increase in xylan digestibility of the material milled for 120 minutes from 54.0% to 72.5% (Table 1). Furthermore, the xylan digestibility of this sample reached 87.7% using the enzyme cocktail supplemented with a 10-fold excess of Optimash BG. These results suggest that the BM-treated xylan has a heterogeneous structure and that its complete degradation requires multiple enzymes.

The main structure of native hemicellulose from eucalyptus is an *O*-acetyl-(4-*O*-methyl-α-D-glucurono)-D-xylan, which is a typical structure for heterogeneous xylan of hardwoods [25]. The heterogeneous xylan from *Eucalyptus globulus *has also been reported to have about one-third of the 4-*O*-methyl-α-D-glucuronopyranosyl residues substituted at *O*-2 by a terminal α-D-galactopyranose [26]. Hemicellulases such as acetylxylan esterase and α-glucuronidase are presumed to play an important role in the degradation of BM-treated eucalyptus xylan, because the *O*-acetyl groups and 4-*O*-methyl-α-D-glucuronopyranosyl residues which sterically hinder xylanase activity have been reported to be removed by these accessory enzymes [27]. The contributing mechanism of Optimash BG to hemicellulose degradation remains unknown. The production of acetate from BM-treated eucalyptus was observed by acetylxylan esterase activity in Acremonium cellulase regardless of the presence or absence of Optimash BG in the enzyme cocktail (data not shown). The structure and composition of hemicellulose is different in hardwoods, softwoods and agricultural residues; therefore, an optimal combination of hemicellulases will be important for the enzymatic hydrolysis of hemicellulose from BM-treated lignocellulosic biomass.

### Effect of HCW treatment on the enzymatic digestibility of eucalyptus

To prepare HCW residues used for combined HCW-BM treatment, HCW treatments were carried out at seven temperature levels in the range of 120 to 240°C for 30 minutes. The yields of sugars in the soluble fraction from HCW treatment as well as the enzymatic hydrolysis yields of the residue are summarized in Table 2. Almost all of the solubilized xylan was found as an oligomer but not as xylose. An increase in the solubilized xylan from HCW-treated eucalyptus was observed above 160°C with increasing CrI of the residue. This suggests that the CrI can be regarded as an indirect measurement of hemicellulose removal from the feedstock: higher values imply more removal of hemicellulose leaving the crystalline cellulose fraction intact in the HCW-treated residues. The highest recovery rate of xylose from the soluble fraction was estimated to be 65.1% at the pretreatment temperature of 180°C (Table 2). The xylose yield from the soluble fraction was severely affected by the generation of decomposition products such as furfural. The amount of furfural in the soluble fraction pretreated at 180°C corresponded to 12.6% of the xylan content in the untreated feedstock, suggesting that more than 77.7% of the xylan was solubilized at this temperature.

**Table 2:** Yields of glucose and xylose recovered from the soluble fraction and residue of eucalyptus pretreated with hot-compressed water

Temperature (°C)	SR^a ^(%)	Yields of hydrolyzed sugars from				Total yield (%)	CrI^b ^(%)
				
		Soluble fraction		Residue			
		Glucose (%)	Xylose (%)	Glucose (%)	Xylose (%)		

120	99.1	0.1	0.0	3.8	0.0	3.1	60.4
140	96.1	0.3	0.7	5.9	5.3	6.1	62.0
160	85.1	0.3	29.9	23.3	21.5	29.3	67.4
180	70.0	1.1	65.1	50.5	9.8	56.4	69.4
200	66.7	2.3	15.7	74.7	4.1	65.2	74.7
220	62.7	1.2	0.0	72.6	1.3	58.8	ND^c^
240	51.8	1.1	0.0	42.5	0.0	34.6	65.6

The glucan and xylan in the soluble fraction were hydrolyzed to monomers with 4% H_2_SO_4_. The hot-compressed water (HCW)-treated residue (1%) was hydrolyzed with an enzyme cocktail (40 FPU/g substrate) for 72 hours. The yields of glucose and xylose are calculated based on the glucan and xylan contents, respectively, in the dry weight of untreated biomass. Total yield of hydrolyzed sugars is calculated from the combined contents of glucan and xylan in the dry weight of untreated biomass.

^a^Solid-remaining yield based on dry weight of HCW-treated residue. ^b^Crystallinity index of the HCW-treated residue. ^c^Not determined.

Increasing the enzymatic digestibility of the HCW-treated residue seems to be related to the removal rate of hemicellulose in the feedstock. Both the enzymatic digestibility and the CrI of the residue were maximal at the pretreatment temperature of 200°C (Table 2). The solid-remaining yield based on dry weight of this residue was 66.7% (Table 2), which contained 51.9% glucan and 1.1% xylan based on the sugar contents in the residue. The data imply that 13.4% glucan and 93% xylan in the untreated feedstock were solubilized under the conditions of 200°C for 30 minutes, but, unfortunately, a large fraction of them seemed to be further degraded. The enzymatic digestibility of this residue with the cellulase loading of 40 FPU/g substrate showed that 86.3% glucan in the residue was hydrolyzed to glucose, corresponding to 74.7% glucan based on sugar contents in the untreated feedstock (Table 2). The residue pretreated at 240°C was found to decrease the CrI as well as the enzymatic digestibility. This is due to a significant loss of glucan content by cellulose solubilization [10].

### HCW-BM treatment of eucalyptus

In this study, we have demonstrated that the BM treatment of eucalyptus provides a favorable substrate for enzymatic hydrolysis and the ethanol fermentation process. The reduction of milling time, with its high energy consumption, is important to utilize the BM treatment as a practical pretreatment for lignocellulosic biomass. As a means of reducing the milling time, we examined the effect of HCW treatment on BM.

**Figure 2:**
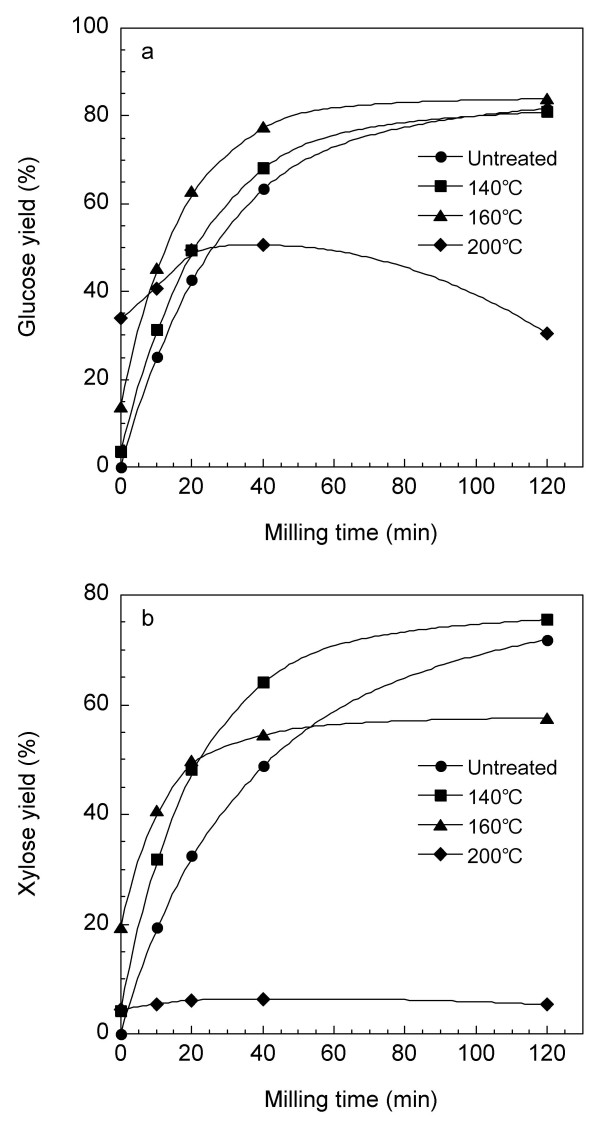
**Effect of milling time on glucan (a) and xylan (b) digestibility of eucalyptus treated with combined hot-compressed water and ball milling**. The residues were pretreated for 30 minutes with hot-compressed water (140-200°C), then dried and milled using ball-milling treatment with pulverisette 7. The pretreated substrates (5%) were hydrolyzed with an enzyme cocktail (4 FPU/g substrate) for 72 hours. Yields of hydrolyzed glucose and xylose are expressed as a percentage of theoretical amount based on the glucan and xylan contents, respectively, in the dry weight of untreated biomass.

The residues pretreated by HCW for 30 minutes were dried and then milled by BM treatment for between 0 and 120 minutes. The HCW-treated residue (160°C, 30 minutes) was found to be effective in elevating the milling efficiency for glucan digestibility. The 40-minute milling of this residue resulted in an approximately three-fold reduction of milling time compared with untreated material (Fig. 2a). BM treatment of the HCW-treated residue (140°C, 30 minutes) was also effective in elevating the milling efficiency for xylan digestibility (Fig. 2b). The structural change in the cellulose-hemicellulose-lignin network by partial solubilization of hemicellulose is presumed to reduce the milling energy. Unexpectedly, a prolonged BM treatment of the HCW-treated residue (200°C, 30 minutes) showed an inhibitory effect on the enzymatic hydrolysis (Fig. 2a). The heat generation or unknown mechanochemical effects during BM treatment of this residue may affect the enzymatic hydrolysis.

### The efficacy of HCW-BM treatment

The efficacy of pretreatment for ethanol production was evaluated by: (i) the total yield of sugars recovered from both glucan and xylan in the feedstock; (ii) the cellulase loading; and (iii) the energy consumption for pretreatment process. Conditions to maximize the total sugar yield are often not the same as those to maximize individual sugar yields [28]. For instance, the highest total sugar yield with the HCW treatment occurred at 200°C for 30 minutes. This corresponded to 65.2% of glucan and xylan contents in the untreated material (Table 2). However, the combined yield of xylose from the soluble fraction and the hydrolyzed residue was only 19.8% due to its further degradation to furfural that is known to be a representative inhibitor of ethanol fermentation. Control of the reaction time with HCW treatment is an important factor in maximizing the solubilization of the hemicellulose and minimizing the further degradation of sugars [11,29]. On the other hand, BM treatment simultaneously increased the enzymatic digestibility of both glucan and xylan. At least 40 minutes of BM treatment was required to obtain a total yield similar to that of the HCW treatment (200°C, 30 minutes). The xylose yield of 49.2% recovered from the 40-minutes milled material was 2.5-fold higher than that from the HCW-treated material (Table 1). Furthermore, the 120-minute milled material showed higher yields of glucose (89.7%) and xylose (72.5%), although the energy requirement for pretreatment was increased.

The total yields of glucose and xylose resulting from both the solubilized fraction by HCW treatment and the residue milled for 20 minutes are summarized in Table 3. The milled residue was hydrolyzed with 4 FPU/g substrate. The CrI of the material pretreated by the combined HCW (160°C, 30 minutes) and BM (20-minute milling) was slightly higher than that pretreated by BM (20-minute milling) alone. However, the combined yield of xylose (79.8%) from this sample was very high, which was similar to that from 120-minute milled material by BM treatment. The combined yield of glucan (63.3%) was also increased compared with that from the material pretreated by BM (20-minute milling) alone. Furthermore, the total yield of sugars (66.7%) was comparable to that from the material pretreated by HCW (200°C, 30 minutes) or BM (40-minute milling), although these samples were hydrolyzed with 40 FPU/g substrate (Tables 1 and 2). The hydrolysate of HCW-BM-treated eucalyptus is expected to have no inhibitory effect on ethanol fermentation, because furfural production by xylose decomposition was barely observed in the HCW treatment at 160°C.

**Table 3:** Yields of glucose and xylose recovered from the soluble fraction and residue of eucalyptus pretreated with combined hot-compressed water and ball milling

Temperature (°C)	Yields of hydrolyzed sugars from				Total yield (%)	CrI^a ^(%)
			
	Soluble fraction		HCW-BM residue			
			
	Glucose (%)	Xylose (%)	Glucose (%)	Xylose (%)		
Untreated	-	-	42.8	32.5	40.7	7.6
140	0.3	0.7	49.4	48.2	49.5	9.1
160	0.3	29.9	63.0	49.9	66.7	13.2
200	2.3	15.7	49.4	6.2	45.6	16.9

The glucan and xylan in the soluble fraction were analyzed as described in Table 2. To prepare combined hot-compressed water (HCW) and ball milling (BM) residues, HCW residues were milled for 20 minutes with pulverisette 7 as described in materials and methods. The HCW-BM residue (5%) was hydrolyzed with an enzyme cocktail (4 FPU/g substrate) for 72 hours. The yields of glucose and xylose are calculated based on the glucan and xylan contents, respectively, in the dry weight of untreated biomass. Total yield of hydrolyzed sugars is calculated from the combined contents of glucan and xylan in the dry weight of untreated biomass. ^a^Crystallinity index of the HCW-BM residue.

**Figure 3:**
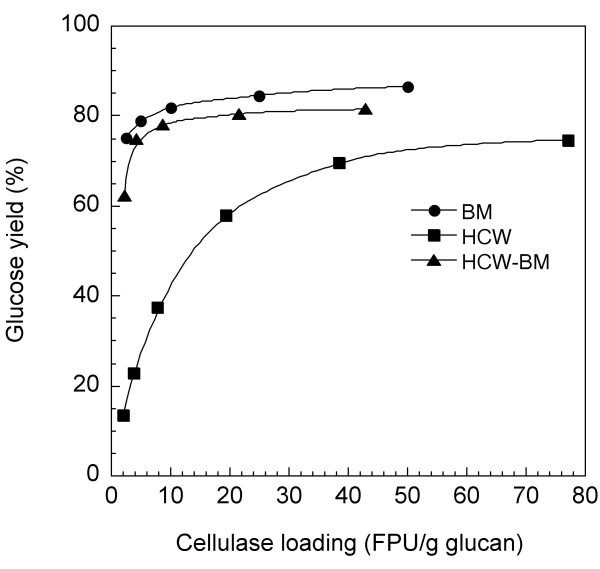
**Effect of cellulase loading on digestibilities of pretreated eucalyptus**. Materials (1%) treated with ball milling (BM; 120-minute milling), hot-compressed water (HCW; 200°C, 30 minutes) and HCW-BM (160°C, 30 minutes; 40-minute milling) were hydrolyzed for 72 hours with enzyme cocktails consisting of 1-40 FPU Acremonium cellulase, 5 IU Novozyme 188 and 0.02 ml Optimash BG per gram of substrate. The digestibilities were compared on the basis of a cellulase loading of FPU per gram of glucan. The glucan contents in the substrates analyzed with 72% H_2_SO_4 _were as follows: HCW (51.9%); BM (40.0%); and HCW-BM (46.6%). The yield of hydrolyzed glucose is expressed as a percentage of the theoretical amount based on the glucan content in the dry weight of untreated biomass.

A comparison of the cellulase loading on glucan digestibility of the HCW-, BM- and HCW-BM-treated samples revealed that the BM- and HCW-BM-treated materials were hydrolyzed with a lower enzymatic loading. When the cellulase loading was evaluated using FPU per gram of glucan content in the substrate, the glucan hydrolysis of the BM- and HCW-BM-treated eucalyptus was accomplished by a cellulase loading of only 10 and 8.5 FPU/g glucan, respectively (Fig. 3). This means that the cost of the enzymatic hydrolysis process is greatly reduced by using the BM treatment.

The disruption of the cellulose-hemicellulose-lignin network structure by the BM treatment seems to be correlated with an increase in the catalytic efficiency of cellulase. The glucan digestibility of HCW-treated residue required a cellulase loading of approximately 40 FPU/g glucan (Fig. 3). Several factors related to a higher CrI of the substrate may contribute to the reduction of digestibility at the lower enzymatic loading: the decrease in the reactivity of the substrate during hydrolysis; different kinds of enzyme inactivation; and non-specific adsorption of enzymes onto lignin [30].

The energy consumption of BM was estimated at 108 MJ/kg wood under the experimental conditions of 100 g wood and 120 minutes of milling. It is known that the milling behavior follows the Rittinger law and that the energy consumption changes according to a scale factor of 0.2 (see [31]). From these data, the energy consumption of ball milling for 120 minutes was calculated at 31 MJ/kg wood under the conditions of a 100 dry t/d bioethanol plant [31]. On the other hand, the energy consumption of the HCW-BM treatment (160°C, 30 minutes; 20-minute milling) was estimated at 6.97 MJ/kg wood under the same conditions, because the energy consumption of HCW treatment was estimated at 1.8 MJ/kg wood. These results suggest that the combined HCW-BM treatment is potentially cost effective: this treatment can reduce not only the cellulase loading for enzymatic hydrolysis but also the energy requirement compared with BM treatment.

It has been reported that multiple pretreatments of wheat straw consisting of BM followed by various chemical treatments were not promising compared with single chemical pretreatments [32]. Our results suggest that the BM treatment should be combined with a pretreatment such as HCW for improving milling time. To the best of the authors' knowledge, the combination of HCW and BM involving mechanochemical effects is a new pretreatment process for lignocellulosic biomass. Although the HCW-treated residue was dried before BM treatment in this study, the drying process causes a significant increase in energy cost for pretreatment. We are performing further studies to establish milling technology with relatively low energy consumption for the wet HCW-treated residue.

## Conclusion

The material pretreated by HCW gave a low yield of xylose accompanying furfural production and a high cellulase loading for glucan hydrolysis. The BM-treated material was a good substrate for enzymatic hydrolysis and subsequent ethanol fermentation, but it required excess energy for milling. We found that a partial solubilization of eucalyptus xylan by the HCW treatment at a relatively low temperature can reduce the milling time by disrupting the cellulose-hemicellulose-lignin network. This indicates that the HCW treatment plays an important role as a non-catalytic pretreatment improving milling efficiency. The combined HCW-BM treatment is proposed as a pretreatment without chemicals that has advantages regarding the reduction in both energy requirement and enzyme loading.

## Methods

### Raw material

Eucalyptus wood chips were kindly supplied by a pulp factory in the vicinity of our research center (Oji Paper Co, Japan). The chips were mixtures of several species of imported eucalyptus. They were milled to pass a 2-mm screen and then collected between 35 and 100 mesh. The materials were homogenized in a single lot to avoid differences in composition among experiments and stored under dry conditions before use. The initial composition of eucalyptus wood was determined to be 40.0% glucan, 10.4% xylan and 28.8% acid-insoluble lignin, according to the analytical procedure recommended by NREL [33].

### HCW treatment

The wood material with a moisture content of 8.8% (2.5 g) and water (30 ml) were mixed in a 50-ml stainless steel autoclave (Nitto Koatsu Co, Tsukuba, Japan) fitted with a two-blade turbine impeller. Argon was purged into the autoclave at an initial pressure of 5.0 MPa. The autoclave was heated and maintained for 30 minutes within ± 2°C of the target temperatures (120-240°C) with stirring. The reaction temperature was monitored using a thermocouple probe, which was inserted into the vessel through an outlet and controlled using proportional-integral-derivative (PID) modules. The heat-up time for pretreatment using this equipment was 14°C/minute. At the end of the reaction period, the autoclave was cooled to room temperature. The solid residue was recovered by filtration, washed with distilled water at room temperature and lyophilized. The soluble fraction was used for high-performance liquid chromatography (HPLC) analysis of monosaccharides and furfural compounds. To determine the total amount of sugars (monomers and oligomers) in the soluble fraction, the samples were hydrolyzed with 4% H_2_SO_4 _at 121°C for 1 hour followed by neutralization and then analyzed with HPLC.

### BM treatment

Untreated or HCW-treated material was dried *in vacuo *at 40°C for 3 days before milling and then subjected to a Planetary Micro mill pulverisette 7 (Fritsch, Germany). The sample (1.0 g) was milled at 400 rpm in a 45-ml milling cup with seven spheres (ϕ = 15 mm). Milling was carried out for a total time of 10-120 minutes (with a cycle of 10-minute milling and 10-minute pausing) at room temperature.

Planetary mill pulverisette 5 (Fritsch, Germany) was used to prepare larger quantities of samples. The sample (25 g) was milled at 250 rpm in a 500-ml milling cup with 25 spheres (ϕ = 20 mm). Milling was carried out for a total time of 240 minutes (with a cycle of 10-minute milling and 10-minute pausing) at room temperature.

### Enzymatic hydrolysis

Enzymatic hydrolysis was performed using an enzyme cocktail constituting 1-40 FPU Acremonium cellulase (Meiji Seika Co, Japan), 0.02 ml Optimash BG (Genencor International, USA), and 5 IU Novozyme 188 (Novozymes, Denmark) per gram of dry substrate. In the standard assay, 1.0 ml of the enzyme cocktail (1-40 FPU/g substrate) in 50 mM acetate buffer (pH 5.0) was added to 0.01-0.05 g of pretreated materials in a 1.5-ml tube. The reaction mixture was incubated at 45°C for 72 hours mixed with a rotator. The experiment was performed in triplicate. In the assay using a high concentration of substrate, 100 ml of the enzyme cocktail (4 FPU/g substrate) in 50 mM acetate buffer (pH 5.0) was added to 0.2-20 g of pretreated materials. The reaction mixture was incubated at 45°C for 72 hour and mixed with a magnetic stirrer. The hydrolysate was centrifuged, and the supernatant was analyzed for glucose and xylose. The acetate production from BM-treated material was assayed using the standard mixture containing 20 mM MES buffer (pH 5.0) instead of 50 mM acetate buffer (pH 5.0).

The filter paper activity (FPU) in Acremonium cellulase and the β-glucosidase activity (IU) in Novozyme 188 were measured according to the standard procedure recommended by the Commission on Biotechnology, IUPAC [34].

Xylanase activities in Acremonium cellulase and Optimash BG were assayed in a reaction mixture (1.0 ml) containing 1% (w/v) birch wood xylan (Sigma-Aldrich, USA), 50 mM acetate buffer (pH 5.0) and appropriately diluted enzyme solutions. After 30 minutes of incubation at 45°C, the reducing sugar liberated was measured using dinitrosalicylic acid. One unit of the enzyme activity is defined as the amount of enzyme that produces 1 μmol of reducing sugar per minute.

### HPLC analyses

Glucose, xylose and acetate were analyzed using a HPLC system equipped with a refractive index detector (RI-2031Plus, JASCO, Japan). Glucose and xylose were analyzed using an Aminex HPX-87P column (7.8 mm I.D. × 30 cm, BioRad, USA) with a Carbo-P micro-guard cartridge. The mobile phase used was doubly deionized water, and the flow rate was 1.0 ml/minute at an 80°C column temperature. Acetate was analyzed using an Aminex HPX-87H column (7.8 mm I.D. × 30 cm, BioRad, USA) with a Cation H micro-guard cartridge. This column was maintained at 35°C, and acetate was eluted with 4 mM H_2_SO_4 _at a flow rate of 0.6 ml/minute.

Furfural and hydroxymethyl furfural were analyzed using a TSK gel ODS-80 Ts QA analytical column (4.6 mm I.D. × 15 cm, TOSOH, Japan) and a photodiode array UV-visible light detector (MD-1510, JASCO, Japan). The mobile phase used was 0.1% phosphate. The furfural compounds were eluted using linier gradient (0 to 6 minutes) with a 1:1 (vol/vol) mixture of 0.1% phosphate and 50% acetonitrile, and their peaks were detected at 275 nm. The flow rate was 1.0 ml/minute with a 40°C column temperature.

### Crystallinity

Crystallinities of the untreated and pretreated wood were measured using a Rigaku RINT-TTR3 X-ray diffractometer (Japan) with Cu Kα radiation at 50 kV and 300 mA. The diffraction spectra were taken using the θ-2θ method. Samples were scanned over the range of 2θ = 2-60° at a rate of 2°/minute. The definition of the CrI is

CrI (%) = [(*I*_002 _- *I*_am_)/*I*_002_] × 100

in which *I*_002 _is the intensity of the crystalline peak at about 2θ = 22.5° and *I*_am _is the intensity at 2θ = 18.7° (see [35]).

## Competing interests

The authors declare that they have no competing interests.

## Authors' contributions

HI carried out the pretreatment and the enzymatic hydrolysis of eucalyptus, and drafted the manuscript. SY and SS designed and coordinated the study and helped to draft the manuscript. TE participated in the process of BM treatment and performed the crystallinity analysis. TS participated in the process of HCW treatment. All authors read and approved the final manuscript.
